# Statistical Degree Distribution Design for Using Fountain Codes to Control the Peak-To-Average Power Ratio in an OFDM System

**DOI:** 10.3390/e24111541

**Published:** 2022-10-26

**Authors:** Cheng Bi, Zheng Xiang, Peng Ren, Ting Yin, Yang Zhang

**Affiliations:** 1 School of Telecommunication Engineering, Xidian University, Xi’an 710071, China; 2 Beijing Electronic Science & Technology Institute, Beijing 100070, China

**Keywords:** fountain codes, orthogonal frequency-division multiplexing, distribution degree, peak-to-average power ratio

## Abstract

Utilizing fountain codes to control the peak-to-average power ratio (PAPR) is a classic scheme in Orthogonal Frequency Division Multiplexing (OFDM) wireless communication systems. However, because the robust soliton distribution (RSD) produces large-degree values, the decoding performance is severely reduced. In this paper, we design statistical degree distribution (SD) under a scenario that utilizes fountain codes to control the PAPR. The probability of the PAPR produced is combined with RSD to design PRSD, which enhances the smaller degree value produced. Subsequently, a particle swarm optimization (PSO) algorithm is used to search the optimal degree value between the binary exponential distribution (BED) and PRSD distribution according to the minimum average degree principle. Simulation results demonstrate that the proposed method outperforms other relevant degree distributions in the same controlled PAPR threshold, and the average degree value and decoding efficiency are remarkably improved.

## 1. Introduction

Orthogonal Frequency Division Multiplexing (OFDM), as a wireless communication scheme, has been widely used in wireless sensor networks, broadcast multicast transmission systems, cooperative relay systems, and deep-space communications systems. Specifically, an OFDM system is essentially a type of multicarrier modulation, which realizes the parallel transmission of high-speed serial data via frequency division multiplexing. It has an intensive ability to resist multipath fading. However, the peak-to-average power ratio (PAPR) is still a challenge in the designed system [1]. When the signal peak exceeds the linear region of the amplifier, signal distortion and mutual interference occur between subcarriers [2,3,4]. In addition, the higher PAPR dilutes the orthogonality between subcarriers and reduces performance in the communication system [5,6,7]. In the last decade, a large number of PAPR reduction schemes have been proposed. Among them, fountain code-based methods are the most attractive.

Luby transform (LT) codes are a type of erasure code with an unlimited rate [8]. The fountain codes only obtain a subset of coded symbols, which is the same as or slightly larger than the source symbol, and the source symbol can easily be recovered. Therefore, the LT codes obtain a performance close to the Shannon limit on the erasure channel. For the entire encoding and decoding process, the encoder in LT codes generates unlimited encoded packages according to the degree distribution, and the decoder accurately restores the original data from any set of encoded packets. Therefore, designing for the degree distribution is capable of enhancing the performance of LT codes. Morteza Hashemi [9] developed feedback-based fountain codes with a dynamically adjusted nonuniform symbol selection distribution, which enhanced the intermediate decoding rate and performance. A poisson distribution (PD) was combined with the robust soliton distribution (RSD) degree distribution by Yao [10], which enhanced the decoding performance. Esa Hyytiä utilized an iterative optimization algorithm to design the degree distribution, from which the idea was borrowed from the importance sampling theory [11].

In this paper, we design statistical degree distribution (SD) under a scenario that utilizes fountain codes to control the PAPR. First, we combine the variation in PAPR probability and the complementary cumulative distribution feature with the RSD to design a PRSD, which enhances the smaller degree value produced. Subsequently, a particle swarm optimization (PSO) algorithm is used to search the optimal degree value between the BED and PRSD according to the minimum average degree principle. Simulations demonstrate that the proposed method performs better than the RSD in decoding while maintaining the same PAPR thresholds.

The remaining parts of the paper are organized as follows: In Section 2, the scheme that utilizes fountain codes to control the OFDM system PAPR and classic degree distribution is introduced. Subsequently, we develop and analyze the proposed statistical degree distribution in Section 3. To verify the effectiveness of the proposed method, the simulation results are given in Section 4. The conclusions are presented in Section 5.

## 2. Materials and Methods

### 2.1. Fountain Codes for the OFDM System PAPR

The signal that uses orthogonal frequency division is superimposed by multiple orthogonal subcarriers under different amplitudes and phases. However, the signal has a noteworthy peak power in the case of large, superimposed subcarriers, which results in mutual interference and distortion between subcarriers [12].

The OFDM signal in the time domain is given as

(1) $$a_{t}=\frac{1}{\sqrt{N}}\overset{N-1}{\underset{n=0}{\sum }}A_{n}e^{j2\pi (\frac{n}{NT})t},0\leq t\leq NT$$

where $A=\left[A_{0},A_{1}\cdots ,A_{N-1}\right]$ denotes an input symbol sequence in the frequency domain, *N* represents subcarriers, *T* represents a period of input symbol, and *NT* is a period of OFDM signal. The signal is generated by summed modulated subcarriers, and each of them is separated by 1/*NT*. In addition, *t* is a continuous time index.

After the Nyquist sampling process, the OFDM signal is expressed as

(2) $$a_{f}=\frac{1}{\sqrt{N}}\overset{N-1}{\underset{n=0}{\sum }}A_{n}e^{j2\pi (\frac{n}{N})f},f=0,1,\ldots ,N-1$$

Therefore, the PAPR of the signal is defined as the ratio of peak power to average power.

(3) $$PAPR=\frac{\underset{0\leq k\leq N-1}{\max}|a(f){|}^{2}}{E\left[|a(f){|}^{2}\right]}$$

where E[·] represents the mathematical expectation of the signal power.

**Figure 1:**
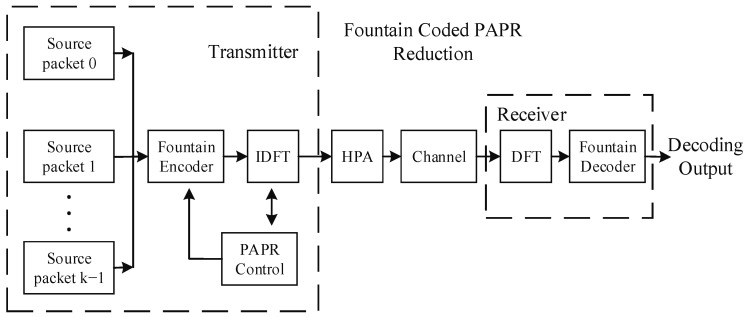
Employing fountain codes to control the PAPR in the OFDM system.

The LT code is a classic fountain code, and reference [8] offers detailed explanations. Reference [12] uses the LT codes to control the PAPR, in which the reduction process in the signal domain transforms into the processing of non-rate-coded packets in the information domain. The representation of the system model is shown in Figure 1.

Figure 1 depicts a typical configuration that employs the fountain codes to control the PAPR in the OFDM system. For simplicity, the guard interval, channel estimation, and synchronization analysis are unnecessary, as they do not affect the process. The receiver is basically synchronized with the transmitter. Moreover, each coded packet contains N symbols on finite fields through N-point inverse discrete Fourier transform (I-DFT) operations for modulations. The PAPR-Control in the block diagram is used to detect whether the PAPR exceeds the threshold y0. If the PAPR exceeds the threshold, the OFDM symbol is discarded. Each data packet has its PAPR value, and the probability is simulated by Equation (3). If the receiver obtains enough encoded packets and decodes successfully, the transmitter terminates encoding and sending packages.

Specifically, the encoding packages are divided into two groups named M1 and M2 in the PAPR controller. The M1 group is composed of packages where the PAPR is less than the predetermined threshold y0, whereas the M2 group owns packages with a higher PAPR threshold. After encoding, the sending process selects packets in the M1 group to send to the receiver and deletes the M2 group. The whole process is depicted in four steps, and reference [12] offers detailed explanations of the process.

Step 1. Calculate source packets for the PAPR and divide them into two groups. The first group consists of packages with PAPRs less than y0, and the others are in the second group, M2.

Step 2. The system has finished encoding and selecting M1 groups to send.

Step 3. The receiver performs the belief propagation (BP) decoding algorithm until the information is completely recovered.

Step 4. When the information is recovered, the fountain code encoder stops transmitting.

### 2.2. LT Codes

The LT code, which was invented by Michael Luby for the Binary Erasure Channel, is a type of linear rateless code based on graph theory. Each code symbol is generated by a linear combination that sets randomly selected information symbols. The research of LT codes consists of encoding, decoding algorithms, and designed degree distribution. The decoding process in LT codes is relatively simple, while the encoding is complex [13,14]. The efficiency of encoding and decoding is significantly improved as the packet lengths increase.

In the encoder, the encoding scheme is equally divided into k groups from the original data, and each code group is generated through the following steps:

Step 1. Select a degree value *d* from the degree distribution set randomly.

Step 2. Select *d* original groups in the k groups randomly.

Step 3. The XOR operation is performed in the original groups to generate encoding groups.

Step 4. The above steps are repeated until decoding is successful.

**Figure 2:**
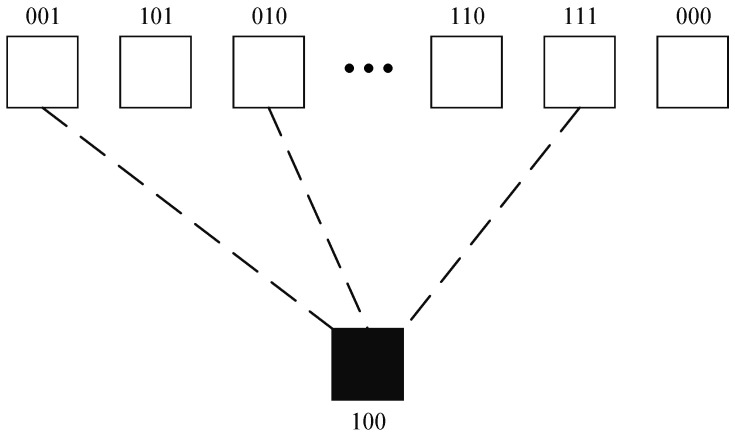
The encoding process for LT.

Figure 2 describes the encoding process with a degree value of 3 (*d* = 3). The empty squares represent the original groups, and the black squares represent the LT encoding groups.

The receiver performs the decoding process after receiving M encoding packets (M > k). In the decoder, a BP or Gaussian elimination algorithm is utilized to recover information. The BP decoding algorithm has the advantage of lower complexity and decoding operation when compared with the GE algorithm [15]. The process of the BP decoding algorithm is described as follows:

Step 1. Find the encoding groups with degree 1 (*d* = 1) and begin the decoding process.

Step 2. The rest of the translated original group makes an exclusive-OR operation with the neighboring group, and it replaces the original code group while deleting its connection relationship.

Step 3. Repeat Step 2 until the decoding is successful.

**Figure 3:**
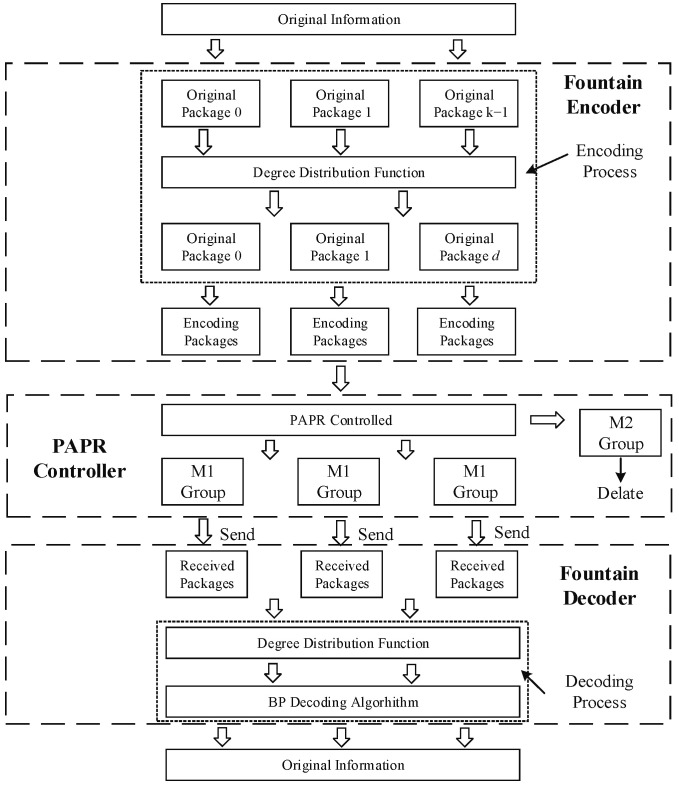
The specific content of the fountain encoder and decoder in utilizing the fountain code to control the PAPR scheme.

The overall system with a fountain encoder and decoder is described in Figure 3. A large number of encoding groups are generated according to the degree distribution function in the encoding process. Similarly, decoding recovery is realized according to the degree of encoding packages and the neighbor relation information. Therefore, the key factor that affects LT code performance is the degree distribution function.

### 2.3. Degree Distribution in LT Codes

Reference [15] describes the typical degree distribution function for LT codes, and we introduce some of them as preparation, which is crucial for further analysis. The classic ideal soliton distribution (ISD) and RSD are defined as follows:

**Definition 1.**  ***ISD*** *The ideal soliton distribution (ISD) distribution definition satisfies*

(4) $$\rho (d)=\{\begin{array}{cc} 1/k, & d=1\\ 1/(d(d-1)), & d=2,3,\ldots ,k \end{array}$$

The LT codes are initially encoded according to the ISD. However, the expected number of degree values 1 is only 1 at the end of the encoding process. The expected number 1 indicates the number of coding symbols connected to only one information symbol among the *k* information symbols in the decoding process, and it is easy to fail in decoding. Therefore, the RSD, which has two parameters *c* and the expected number *R* is developed, and it adjusts the degree 1 in the decoding. Luby proposes τ(d) and adds *R* to it to generate the RSD.

**Definition 2.**  ***RSD*** *The RSD definition satisfies*

(5) $$\tau (d)=\{\begin{array}{c} \begin{array}{cc} \frac{R}{dk}, & d=1,2,\ldots ,k/R-1 \end{array}\\ \begin{array}{cc} R\ln(R/\delta )/k, & d=k/R \end{array}\\ \begin{array}{cc} 0, & d=k/R+1,\ldots ,k \end{array} \end{array}$$

where $R=c\sqrt{k}\ln(k/\delta )$, *c* is a constant with c>0, and δ represents the probability of decoding failure with δ∈[0,1]. The RSD is composed of ρ(d) and τ(d), and function is defined as follows:

(6) $$\beta =\overset{k}{\underset{d=1}{\sum }}\rho (d)+\tau (d)$$

(7) $$RSD=\frac{\rho (d)+\tau (d)}{\beta },\;\mathrm{for}\;d=1,\ldots ,k$$

Similarly, the BED that enhances the efficiency in the initial stage of the decoding process is defined as follows [14].

**Definition 3.**  ***BED*** *The BED definition satisfies*

(8) $$\delta (d)=\{\begin{array}{cc} \frac{1}{16e}, & d=1\\ \frac{1}{2^{d-1}}, & d=2,\ldots ,k \end{array}$$

(9) $$BED=\frac{\delta (d)}{\sum_{d=1}^{k}\delta (d)},d=1,2,\ldots ,k$$

It is worth noting that the degree distribution design must obey three characteristics.

1. The ratio of degree value 2 should be the largest. When *k* → ∞, the ratio should be close to 1/2.
2. The ratio of degree 1 is smaller than that of degree 2, and greater than 0.
3. The average degree determines the complexity of the code and should be as small as possible.

## 3. Algorithm Development for Statistical Distribution

In this section, we follow the three characteristics and redesign the statistical degree distribution based on the probability of the PAPR threshold.

### 3.1. Design for PRSD

The degree distribution function of fountain codes not only determines the complexity of encoding and decoding but also affects the success rate of decoding. Because the M2 group is deleted during the transmission process, the receiver remarkably increases the decoding cost when the system has a small, controlled threshold. Therefore, the statistical characteristic of the PAPR is used in combination with the RSD, which increases the probability of a smaller degree value. The statistical characteristic of the PAPR typically uses the statistics cumulative distribution function (CDF) and CCDF to express, and the relationship between CDF and CCDF is given as:

(10) CCDF=1−CDF

Because the amplitude of the OFDM symbol obeys the Rayleigh distribution, its power y obeys the $\chi^{2}$ distribution in freedom with mean value 0 and variance 1. Therefore, the CDF function is given as:

(11) $$\mathrm{Pr}\left(y\leq y_{0}\right)=\int_{0}^{PAPR_{0}}e^{-y}dy=1-e^{-y_{0}}$$

The key factor in the design process is the controlling process, which maintains that the probability of degree 1 is close to $\ln(k/\delta )*\sqrt{k}$. The probability of decoding failure δ represents the probability that *k* deviates from the mean of the ripple length. The PAPR controlling process deletes a large number of encoding packets in the M2 groups when threshold y_0_ is set low. To decode successfully, more encoding packages with degrees 1 and 2 are required in the decoding process. However, RSD limitedly generates degree 1, 2, and other smaller degree values with insufficient encoding packages; the decoding cost increases strikingly. Therefore, we consider optimizing the RSD by using the statistical characteristics of the PAPR. Equation (11) decreases the probability density greater than the threshold as the threshold increases. We define *m* to represent the probability that the symbol power is less than the threshold, and the CCDF function is combined with the RSD. The deviation degree of the smaller degree value is obviously less than that of the larger degree value. Therefore, the production of smaller degrees is higher than that of large degrees under the same decoding failure probability *δ*.

In addition, we designate the degree distribution as PRSD. The variation of function *τ*(*d*) is redefined as:

(12) $$\tau (d)=\{\begin{array}{cc} mR/dk, & d=1,2,\ldots ,D-1\\ mR\ln(R/\delta )k, & d=D \end{array}$$

where *R* represents $c*\ln(k/\delta )\sqrt{k}$, and *D* = [*k*/R].

### 3.2. Statistical Degree Distribution Designed

The ripple is the set of degree 1 during LT decoding, which reflects the connection between decoding performance and degree distribution. The BED has a large expectation ripple in the initial stage, and it reduces the failure probability of decoding. The probability of BED degree production is decreased with the increase of *k* in Equation (9). Because the performance in the decoding and encoding process is determined by degree values 1 and 2, the BED initially achieves a remarkable decoding success rate.

Therefore, the performance advantages of PRSD and BED are combined in the proposed method, and we regard the D-dimensional parameter quantity generated by PRSD and BED as the solution in the D-dimensional space. The statistical degree distribution is obtained in the solution space via PSO according to the criterion in which the average degree value is the lowest. The results of each particle in the solution space represent the value of the degree distribution. The initial value of each degree ratio is from the PRSD and BED, and the range is defined from the maximum and minimum values of the BED and PRSD.

(13) Ω(d)∈[min(ζ(d),μ(d)),max(ζ(d),μ(d))],d=1,2,…,k

where *ζ*(*d*) is the value of the BED, and *μ*(*d*) represents the PRSD.

The objective function is given.

(14) $$\bar{c}=\overset{D}{\underset{d=1}{\sum }}c_{d}*d,d=1,2,\ldots ,k$$

where $\bar{c}$ is the average degree, and $c_{d}$ represents the probability of each degree value. The constraint of the function is designed as follows:

(15) $$\overset{k}{\underset{d=1}{\sum }}c_{d}=1,c_{d}⩾0$$

The process of searching each particle is described.

(16) $$v_{IJ}^{\left(s+1\right)}=w_{1}v_{IJ}^{\left(s\right)}+c_{1}r_{1}\left(p_{IJ}^{\left(s\right)}-y_{IJ}^{\left(s\right)}\right)+c_{2}r_{2}\left(p_{GJ}^{\left(s\right)}-y_{IJ}^{\left(s\right)}\right)$$

(17) $$y_{IJ}^{\left(s+1\right)}=y_{IJ}^{\left(s\right)}+v_{IJ}^{\left(s+1\right)}$$

where *I* is the number of particles; *J* is the dimension of the search space; *s* is the number of iterations; *c*1 and *c*2 are constant, and *r*1 and *r*2 are random numbers between 0 and 1; $v_{IJ}^{}$ represents the velocity of particles; $y_{IJ}^{}$ are locations for particles. Similarly, $p_{IJ}^{}$ and $p_{GJ}^{}$ represent the best location for a single particle and population, respectively. Additionally, the velocity and position of each particle are updated according to Equations (16) and (17), respectively. The design process of the statistical degree distribution is described in Table 1.

## 4. Simulations

In this section, the performance of the decoding cost is evaluated in terms of different conditions. The simulations deploy 100, 400, and 800 original packages that are analyzed in detail under the same PAPR threshold. The proposed method is compared with RSD and Poisson-moved-robust soliton distribution (PM-RSD) which was designed by Zhang [16], and we set the parameter *δ* to 0.1, *c* to 0.3, and the number of system subcarriers from 8 to 32. Similarly, the BPSK modulation and belief propagation algorithm are applied for the encoding and decoding process. To improve the efficiency of the simulation, we limit the cost to thirty times for the original packets. In addition, the parameters *c*1 and *c*2 are set to 2 in the PSO algorithm. Similarly, the maximum number of iterations is 200, and the dimensions are set to 100, 400, and 800.

**Figure 4:**
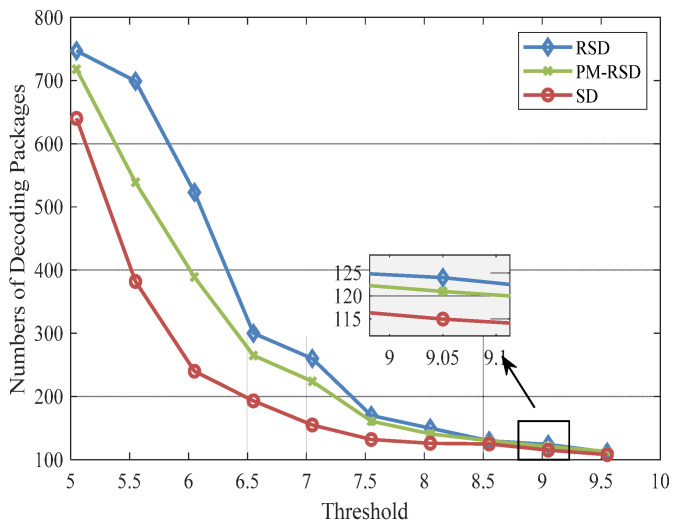
Different degree distribution performance with decoding cost.

The condition in which the number of 100 original packages and 32 subcarriers are used is evaluated for decoding performance. The simulations are performed 100 times, and the results are averaged for analysis. In addition, the range of the threshold is set from 5.05 to 9.5 in the PAPR controlling process, and the comparison of the proposed SD, RSD, and PM-RSD are revealed in Figure 4.

Figure 4 depicts the number of encoding packets sent after successful decoding. The minimum average degree value of the proposed method is 4.91, where probabilities of degrees 1 and 2 are 0.045 and 0.498, respectively. It is evident from the results that the proposed method performed better than RSD, and it reduced 152 packages under the threshold of 7. The maximum number of decoding required with RSD, MRSD, and the proposed method is 747, 725, and 640, respectively, when the controlling threshold is set to 5.05. The variation of decoding cost is basically stabilized when the PAPR threshold is over 8.5, and the performance of different degree distributions gradually converges when the threshold exceeds 9. In addition, the proposed method has remarkable performance at lower thresholds owing to the smaller degree values increased.

**Figure 5:**
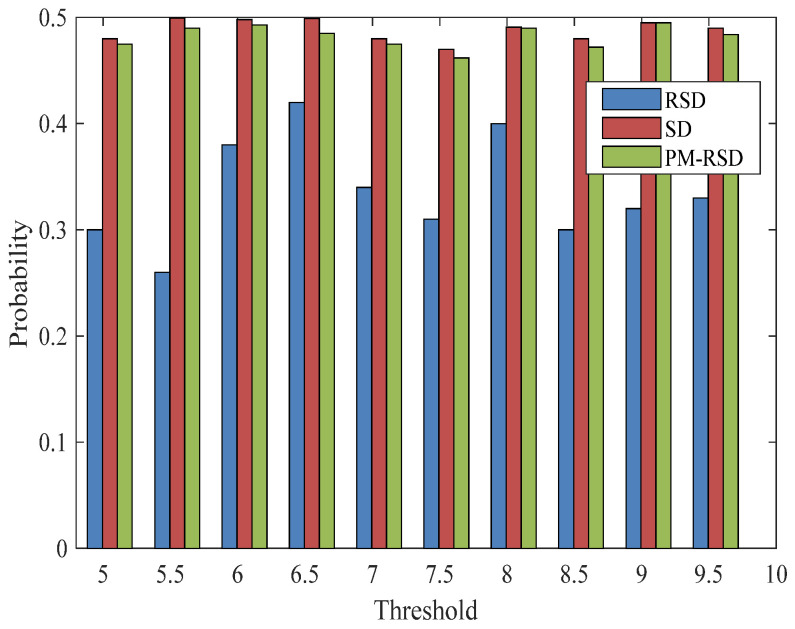
Degree distribution performance with degree 2.

The probability of degree 2 is indicated in Figure 5 under the above condition. There is a probability of 0.498 and 0.475 in the maximum and minimum, respectively, with the proposed method applied, and PM-RSD is 0.491 and 0.462, respectively. Similarly, the probability of degree value 2 is significantly enhanced in the proposed method compared with RSD under different PAPR thresholds. The maximum distinction is 0.188 for the probability between RSD and the proposed method under the threshold of 5.5. In addition, the probability of degree 1 is 0.045 and 0.033 under the proposed method and RSD, respectively.

**Figure 6:**
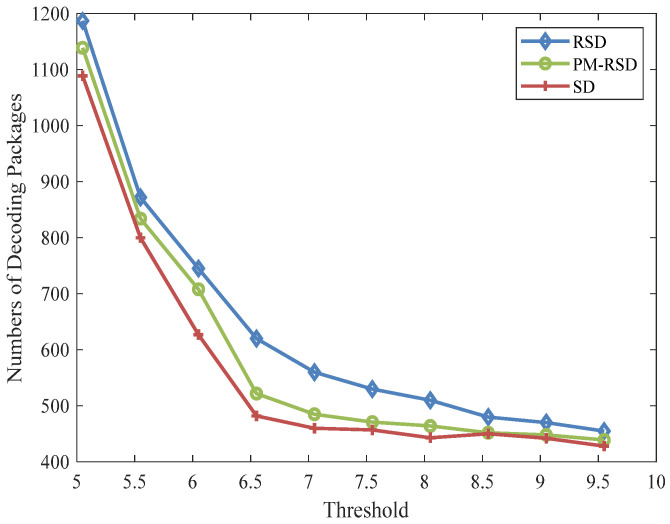
Different degree distribution performances with successful decoding.

The performance of the proposed method, along with the RSD and PM-RSD, are presented in Figure 6. The parameters in the original encoding packets and subcarriers are set to 400 and 16, respectively, and *δ* and *c* stay the same as the above simulation. Similarly, the parameters of the PSO algorithm are consistent with the previous results. It is clear in Figure 5 that the proposed method performed well. The maximum distinction of encoding packages is 140 between the RSD and the proposed method under the threshold of 6.5. The maximum number of decoding packages is 1094 in the proposed method when the threshold is 5, whereas the minimum number of packages is 418 with a threshold of 9.5. Conversely, the maximum and minimum decoding packages are 1198 and 453, respectively, in the RSD. Similarly, the average degree and packages in Table 2 reveal that the proposed method performed well compared with RSD and PM-RSD.

**Figure 7:**
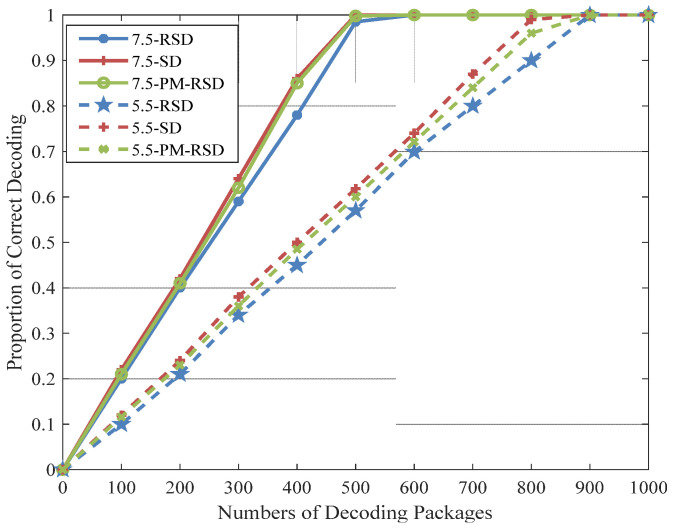
Proportion of correct decoding for different degree distributions.

The rate of decoding is illustrated in Figure 7, and the threshold is set to 5.5 and 7.5 during 400 original packages. It is worth noting that the success rate in the initial decoding of the proposed method is better than the RSD and PM-RSD on account of combined BED. Similarly, the proposed method performs better with the same decoding rate when compared with RSD and PM-RSD.

The average number, average degree, maximum number, and minimum number in the proposed method and other distributions considered in the comparison are summarized in Table 2, Table 3 and Table 4. Similarly, the average number, average degree, maximum number, and minimum number in the proposed method and other distributions considered in the comparison are summarized in Table 3 and Table 4.

Table 4 reveals that the maximum decoding packages in the proposed method are reduced by 995 when compared with RSD. The proposed degree distribution performs well in average degree and decoding cost compared to RSD and PM-RSD. Simulations and results demonstrate that the proposed method has reduced the cost of decoding when the same PAPR threshold is controlled.

## 5. Conclusions

In this paper, we develop a statistical degree distribution based on the probability of the PAPR produced using fountain codes to control the PAPR scheme. Specifically, we combine BED to enhance the rate of initial decoding success. The proposed statistical degree distribution enhances the smaller degree value produced, especially with degrees 1 and 2. Simulation results show that the proposed statistical degree distribution outperforms RSD and PM-RSD when maintaining the same controlled PAPR threshold. In addition, the performance of the average degree and decoding cost has improved. The proposed method provides fresh insight into the designed degree distribution.

## Figures and Tables

**Table 1:** The architecture of the statistical degree distribution.

Degree Distribution Design for LT Codes	
1	$\mathrm{Set}\;y\to y_{0}$ as the threshold for PAPR
2	Calculate BED
3	$\mathrm{for}\;y_{0}\in {\mathrm{PAPR}}_{\mathrm{threshold}}$ do
4	y_PAPR_ ← Calculate CCDF for PAPR
5	Update *τ*(*d*)
6	Calculate max(ζ(d),μ(d)) and min(ζ(d),μ(d))
7	Optimate between PRSD and BED
8	Update degree distribution
9	End for

**Table 2:** k = 100 *N* = 32 Comparison of different degree distributions.

Degree Distribution	Average Number	Average Degree	Maximum Number	Minimum Number
RSD	202.40	5.59	747	111
PM-RSD	195.27	5.42	725	108
SD	189.58	4.91	640	106

**Table 3:** k = 400 N = 16 Comparison of different degree distributions.

Degree Distribution	Average Number	Average Degree	Maximum Number	Minimum Number
RSD	624.57	9.70	1198	453
PM-RSD	565.48	7.40	1165	431
SD	551.26	6.74	1094	418

**Table 4:** k = 800 N = 8 Comparison of different degree distributions.

Degree Distribution	Average Number	Average Degree	Maximum Number	Minimum Number
RSD	1422.18	13.63	2850	835
PM-RSD	1345.82	11.58	2473	829
SD	1183.56	10.06	1865	805

## Data Availability

The data used to support the findings of this study are available from the corresponding author upon request.

## Nomenclature

**Number**	**Notations and Symbols**	**Explanation**
1	$a_{t}$,$a_{f}$	OFDM signal
2	$A_{n}$	Input symbol sequence in the frequency domain
3	*N*	Subcarriers
4	*t*	Continuous time index
5	*T*	A period of an input symbol
6	E[·]	The mathematical expectation of signal power
7	*d*	Degree value
8	*k*	Groups from the original data
9	$y_{0}$	PAPR threshold
10	*y*	PAPR value
11	δ	Probability of decoding failure
12	*c*	Constant
13	*m*	The probability that the symbol power is less than the threshold
14	$\delta_{d}$	Degree for BED distribution
15	$\mu_{d}$	Degree for PRSD distribution
16	$\bar{c}$	Average degree
17	$c_{d}$	The probability of each degree value
18	*c*1, *c*2, *r*1, *r*2	The PSO algorithm parameters
19	$v_{IJ}$	Velocity for particles in the PSO algorithm
20	$y_{IJ}$	Location for particles in the PSO algorithm
21	$P_{IJ}$	The best location for a single particle in the PSO algorithm
22	$P_{GJ}$	The best location for the population in the PSO algorithm
